# Comparative clinical outcomes of suprapatellar intramedullary nailing vs. minimally invasive plate osteosynthesis fixation for distal tibial fractures: a retrospective cohort study

**DOI:** 10.3389/fsurg.2026.1869900

**Published:** 2026-06-17

**Authors:** Bing Song, Qiuxue Zhang, Jize Qian

**Affiliations:** 1 Department of Orthopedic, Qilu Hospital Dezhou Hospital, Shandong University, Dezhou, Shandong, China; 2 Department of Interventional Medicine, Qilu Hospital Dezhou Hospital, Shandong University, Dezhou, Shandong, China

**Keywords:** complications, distal tibial fracture, fracture healing, functional outcomes, intramedullary nailing, minimally invasive plate osteosynthesis, suprapatellar approach

## Abstract

**Introduction:**

Distal tibial fractures are challenging injuries due to limited soft tissue coverage and complex biomechanics. Suprapatellar intramedullary nailing (IMN) and minimally invasive plate osteosynthesis (MIPO) are widely used; however, their comparative effectiveness remains controversial. This study compared perioperative parameters, functional outcomes, radiographic results, and complications to inform surgical decision-making.

**Methods:**

This retrospective cohort study included 70 patients with distal one-third tibial fractures treated with suprapatellar IMN (*n* = 35) or MIPO (*n* = 35) between January 2021 and December 2024. Perioperative data, functional outcomes (VAS, AOFAS, Lysholm, and ROM), radiographic results, and complications were assessed during a 12–18 month follow-up. Multivariate logistic regression identified independent risk factors for complications.

**Results:**

The IMN group had shorter operative time, less blood loss, and smaller incisions (all *P* < 0.001), whereas the MIPO group required fewer fluoroscopic exposures (*P* < 0.001). At 1 and 3 months, IMN showed superior VAS, AOFAS, Lysholm scores, and knee ROM (*P* < 0.05), with no differences at 6 and 12 months. Time to union was shorter in the IMN group (21.8 ± 2.6 vs. 23.7 ± 2.9 weeks, *P* = 0.005). Alignment was comparable, although MIPO showed a trend toward better control. Anterior knee pain was more common after IMN, while incision-related complications were more frequent after MIPO. Fracture classification, surgical method, operative time, and BMI were independent risk factors.

**Conclusion:**

Both techniques are effective. IMN favors early recovery and fracture healing, whereas MIPO offers better alignment control. Surgical strategy should be individualized.

## 1 Introduction

Distal tibial fractures are a common type of long bone fracture, accounting for approximately 10%–13% of all tibial fractures (1, 2). Given the unique anatomical characteristics of this region, including its limited soft tissue coverage, relatively poor blood supply, and its location within the metaphyseal–diaphyseal transition zone, these fractures often involve varying degrees of comminution and articular participation (3–5). Therefore, management of distal tibial fractures requires not only restoration of anatomical alignment and the lower limb mechanical axis, but also preservation of the local blood supply, to minimize complications, such as infection, delayed union, and nonunion (6, 7). However, due to the complex biological and biomechanical environment, surgical treatment of distal tibial fractures remains challenging (8).

Currently, surgical intervention is the mainstay of treatment for these fractures, with intramedullary nailing (IMN) and minimally invasive plate osteosynthesis (MIPO) being the most commonly used fixation techniques (9–11). As a typical intramedullary fixation method, IMN offers several advantages, including minimal surgical trauma, limited soft tissue disruption, alignment with the physiological load-bearing axis, and facilitation of early functional rehabilitation (12, 13). In particular, development of the suprapatellar IMN approach in recent years has partially overcome the limitations of conventional infrapatellar nailing, such as difficulty in fracture reduction and inadequate control of alignment in distal fractures, thereby expanding the indications of this fixation approach (14, 15).

On the other hand, the MIPO approach is based on the concept of biological fixation and aims to preserve the blood supply at the fracture site by using bridging fixation techniques (16). MIPO has demonstrated favorable clinical outcomes, particularly in comminuted and complex fractures, and is considered advantageous for maintaining limb alignment (17).

Nevertheless, considerable controversy exists regarding the relative merits of IMN and MIPO fixation in the treatment of distal tibial fractures. Some studies have suggested that IMN is superior in reducing surgical trauma, promoting fracture healing, and facilitating early functional recovery, whereas others have reported that MIPO provides better control of alignment and reduces the risk of angular deformity (9–11, 17). Moreover, the two techniques differ in their complication profiles: anterior knee pain has been associated with IMN, while wound-related complications have been associated with MIPO fixation (18). Despite extensive research, currently available evidence remains inconsistent, and most studies have been limited by small sample sizes or the potential selection bias inherent to retrospective designs (9, 10). Furthermore, comprehensive analyses based on real-world clinical data are lacking.

Therefore, in order to provide further evidence for the rational selection of surgical strategies for treating distal tibial fractures in clinical practice, we here retrospectively compared the clinical data of 70 patients with distal tibial fractures who were treated via either IMN or MIPO fixation at our institution between January 2021 and December 2024. Specifically, we compared the perioperative parameters, functional outcomes, radiographic results, and complication rates between these two approaches.

## 2 Materials and methods

### 2.1 Study design and ethics

This was a single-center retrospective study. Seventy patients with distal tibial fractures who were treated at our institution between January 2021 and December 2024 were retrospectively selected according to particular inclusion and exclusion criteria. During the study period, the surgical techniques and perioperative management protocols remained essentially unchanged. Patients were divided into two groups based on the surgical method used: suprapatellar IMN (the IMN group) or MIPO fixation (the MIPO group). The choice of surgical technique was not randomized but was determined through routine clinical decision-making based on fracture characteristics, soft tissue condition, surgeon preference and experience, and patient-specific factors. In general, suprapatellar IMN was preferentially considered for patients with relatively simple extra-articular fractures and satisfactory soft tissue conditions, particularly when early mobilization was desired. Conversely, MIPO fixation was more frequently selected for fractures with greater metaphyseal instability, concerns regarding alignment control, or situations in which the surgeon considered plate fixation to provide more stable distal support. During the study period, all eligible patients meeting the inclusion criteria were consecutively screened and retrospectively assigned to the IMN or MIPO group according to the actual surgical procedure performed. This study was approved by the institutional ethics committee and was conducted in accordance with the Declaration of Helsinki.

### 2.2 Inclusion and exclusion criteria

Patients were included if they met the following criteria: distal one-third tibial fracture classified as AO types 42-A, 42-B, or 43-A, with the fracture center located 4–11 cm proximal to the distal tibial articular surface; closed fractures or Gustilo type I open fracture; treatment with either suprapatellar IMN or MIPO fixation following closed reduction; unilateral fresh fracture at initial presentation; and availability of complete clinical and follow-up data. Patients were excluded if they had multiple trauma or pathological fractures; were younger than 18 years or older than 65 years; or had comorbidities that could affect wound healing, fracture union, or limb function, such as diabetes mellitus or hemiplegia.

### 2.3 Surgical procedures

#### 2.3.1 IMN group

All procedures were performed under combined intravenous and inhalation anesthesia with the patient in the supine position. The hip on the affected side was slightly elevated, and a support pad was placed under the lower leg to maintain the knee in 20°–30° of flexion. A tourniquet was applied, and standard sterile preparation and draping were performed. For associated distal fibular fractures affecting ankle stability, open reduction and internal fixation were performed first, while proximal fibular fractures were not routinely treated. A 2–3 cm longitudinal incision was made approximately two fingerbreadths proximal to the superior pole of the patella. The skin, fascia, and quadriceps tendon were incised layer by layer to access the knee joint cavity. Under fluoroscopic guidance, an entry point was established at the medial edge of the lateral tibial spine on the anteroposterior view and at the junction of the tibial plateau and anterior slope on the lateral view. A guidewire was inserted into the medullary canal, and traction was applied to achieve fracture reduction. Sequential reaming was performed, followed by insertion of an appropriately sized suprapatellar intramedullary nail. Proximal and distal interlocking screws were placed in multiple planes, and an end cap was inserted. A drainage tube was placed in the joint cavity, followed by irrigation and wound closure.

#### 2.3.2 MIPO group

All procedures were performed under the same anesthesia and positioning. A tourniquet was applied, and standard sterile preparation was conducted. Distal fibular fractures affecting ankle stability were fixed first. A 2–3 cm curved incision was made over the medial malleolus. A subcutaneous tunnel was created between the periosteum and soft tissue using a periosteal elevator. A locking plate was inserted retrogradely along the anteromedial aspect of the tibia. Two Kirschner wires were temporarily placed through distal holes for positioning, and fluoroscopy was used to confirm proper plate placement and to avoid intra-articular screw penetration. Fracture reduction was achieved with traction and, if necessary, clamp assistance. After confirming alignment and plate position, one cortical screw was placed proximally and one distally, followed by insertion of 3–4 locking screws through small incisions. Final fluoroscopy was used to confirm satisfactory reduction, implant position, and screw length. A subcutaneous drain was placed, followed by irrigation and wound closure.

### 2.4 Postoperative management

All patients were managed postoperatively by using a standardized rehabilitation protocol. Active ankle and knee range-of-motion (ROM) exercises were initiated within 24–48-hours postoperatively. Weight-bearing was gradually increased, based on radiographic healing, with partial weight-bearing typically initiated at 4–6-weeks postoperatively, progressing to full weight-bearing after fracture union.

### 2.5 Outcome measures

Baseline demographic and clinical variables, including age, sex, body mass index (BMI), smoking history, alcohol consumption, mechanism of injury, fracture classification, and soft tissue condition, were collected and compared between groups to assess baseline comparability and potential confounding factors. The primary outcomes were functional recovery assessed by the American Orthopedic Foot and Ankle Society (AOFAS) score, Lysholm knee score, and knee ROM. Secondary outcomes included perioperative parameters, radiographic outcomes, and postoperative complications. Perioperative parameters included operative time, total incision length, intraoperative blood loss, fibular fixation status, number of fluoroscopic exposures, time to ambulation, and length of hospital stay. Functional outcomes included time to full weight-bearing, visual analogue scale (VAS) for pain, Lysholm knee score, and AOFAS score, assessed at 1-, 3-, 6-, and 12-months postoperatively (final follow-up). Knee and ankle ROM were also measured. Radiographic assessments included limb length discrepancy, lateral distal tibial angle (LDTA), and anterior distal tibial angle (ADTA), as well as time to fracture union. All radiographic measurements were independently performed by two experienced orthopedic surgeons who were blinded to group allocation, and the mean values were used for analysis to improve reliability. Fracture union was defined as radiographic evidence of a bridging callus across at least three cortices, combined with painless full weight-bearing. Delayed union was defined as failure to achieve union by 6-months postoperatively, and nonunion was defined as absence of healing progression by 9-months postoperatively.

### 2.6 Statistical analysis

Statistical analyses were performed using SPSS version 26.0 (IBM Corp., Redmond, WA, USA). Categorical data are presented as counts and percentages. Continuous variables are expressed as mean ± standard deviation (x¯ ± s), and normality of data distribution was assessed using the Shapiro–Wilk test. No missing data were identified for key variables; therefore, no data imputation was performed. In univariate analysis, continuous variables were compared using independent-samples *t*-tests or nonparametric tests, as appropriate, while categorical variables were analyzed using the chi-square test or Fisher's exact test. Ordinal data were analyzed using rank-sum tests. Variables with *P* < 0.05 in univariate analysis were entered into multivariate logistic regression analysis. Multicollinearity was assessed using the variance inflation factor, and model fit was evaluated using the Hosmer–Lemeshow goodness-of-fit test. Two-sided *P* values < 0.05 were considered statistically significant. Effect sizes were reported as mean differences (MDs) with corresponding 95% confidence intervals (CIs) for continuous variables and odds ratios (ORs) with 95% CIs for categorical outcomes where appropriate, to improve the interpretability and clinical relevance of statistical findings.

## 3 Results

### 3.1 Perioperative outcomes

All patients successfully underwent surgery without any serious perioperative complications. Baseline demographic and injury-related characteristics are summarized in Table 1. No statistically significant differences were observed between the two groups regarding age, sex, BMI, smoking history, alcohol consumption, mechanism of injury, fracture classification, or soft tissue condition (all *P* > 0.05), indicating satisfactory baseline comparability. The IMN group demonstrated a significantly shorter operative time than the MIPO group [mean difference [MD] = −18.6 min, 95% confidence interval [CI]: −25.4 to −11.8, *P* < 0.001]. The IMN group also had significantly lower intraoperative blood loss (MD = −46.1 mL, 95% CI: −59.7 to −32.5, *P* < 0.001). In addition, the total incision length in the IMN group was significantly smaller than that in the MIPO group (*P* < 0.05). Conversely, the number of intraoperative fluoroscopic exposures was significantly lower in the MIPO than in the IMN group (*P* < 0.05). No statistically significant differences between the two groups in terms of fibular fixation rate, time to ambulation, or length of hospital stay were observed (*P* > 0.05). Detailed results are presented in Table 2.

**Table 1:** Baseline characteristics of patients in the IMN and MIPO groups.

Variable	IMN group (*n* = 35)	MIPO group (*n* = 35)	t/*χ*² value	*P* value
Age (years)	46.8 ± 11.5	48.2 ± 10.9	0.53	0.598
Male sex, *n* (%)	22 (62.9%)	24 (68.6%)	0.26	0.611
BMI (kg/m²)	25.1 ± 3.2	25.8 ± 3.5	0.87	0.389
Smoking history, *n* (%)	12 (34.3%)	13 (37.1%)	0.06	0.804
Alcohol consumption, *n* (%)	9 (25.7%)	10 (28.6%)	0.08	0.775
Fracture side (left), *n* (%)	18 (51.4%)	16 (45.7%)	0.23	0.633
Mechanism of injury			0.71	0.701
Traffic accident	15 (42.9%)	17 (48.6%)		
Fall from height	11 (31.4%)	10 (28.6%)		
Other trauma	9 (25.7%)	8 (22.8%)		
AO/OTA fracture classification			0.58	0.749
42-A	11 (31.4%)	10 (28.6%)		
42-B	12 (34.3%)	11 (31.4%)		
43-A	12 (34.3%)	14 (40.0%)		
Gustilo type I open fracture, *n* (%)	6 (17.1%)	7 (20.0%)	0.09	0.759
Soft tissue condition (Tscherne grade)			0.47	0.791
Grade 0	14 (40.0%)	13 (37.1%)		
Grade I	13 (37.1%)	15 (42.9%)		
Grade II	8 (22.9%)	7 (20.0%)		

Data are presented as mean ± standard deviation or *n* (%), unless otherwise stated. BMI, body mass index; IMN, intramedullary nailing; MIPO, minimally invasive plate osteosynthesis; AO/OTA, Arbeitsgemeinschaft für Osteosynthesefragen/Orthopaedic Trauma Association.

**Table 2:** Comparison of perioperative outcomes between the two groups.

Group	*n*	Operative time (min)	Blood loss (mL)	Incision length (cm)	Fluoroscopy times (*n*)	Fibular fixation *n* (%)	Time to ambulation (days)	Length of hospital stay (days)
IMN group	35	85.6 ± 12.3	92.5 ± 25.4	3.1 ± 0.6	18.7 ± 4.2	14 (40.0%)	3.8 ± 1.1	9.6 ± 2.3
MIPO group	35	104.2 ± 15.7	138.6 ± 30.8	6.8 ± 1.2	13.5 ± 3.1	16 (45.7%)	4.2 ± 1.3	10.3 ± 2.6
*t*/χ² value	—	5.52	7.05	16.20	6.01	0.23	1.45	1.21
*P* value	—	<0.001	<0.001	<0.001	<0.001	0.633	0.152	0.231
Effect size (95% CI)	—	MD = −18.6 (−25.4 to −11.8)	MD = −46.1 (−59.7 to −32.5)	MD = −3.7 (−4.2 to −3.2)	MD = 5.2 (3.2 to 7.2)	OR = 0.79 (0.29 to 2.15)	MD = −0.4 (−0.97 to 0.17)	MD = −0.7 (−1.85 to 0.45)

Data are presented as mean ± standard deviation or *n* (%). MD, mean difference (IMN group minus MIPO group); OR, odds ratio; CI, confidence interval.

IMN, intramedullary nailing; MIPO, minimally invasive plate osteosynthesis.

### 3.2 Follow-up outcomes

All patients completed follow-up, with a follow-up duration ranging from 12 to 18 months (x¯ ± s: 14.2 ± 2.1 months). At 1 and 3 months postoperatively, the IMN group demonstrated significantly lower VAS scores than the MIPO group. At 1 month, the mean difference (MD) was −0.9 points [95% confidence interval (CI): −1.31 to −0.49, *P* < 0.001], whereas at 3 months, the MD was −0.7 points (95% CI: −1.06 to −0.34, *P* < 0.001). However, no statistically significant differences were observed between the two groups at 6 and 12 months postoperatively (*P* > 0.05). However, no significant differences were observed between the two groups at 6- and 12-months postoperatively (*P* > 0.05). In terms of function, both the AOFAS and Lysholm knee scores improved progressively over time in both groups and reached a plateau after 6-months postoperatively. At 1 and 3 months postoperatively, the IMN group demonstrated significantly higher AOFAS scores than the MIPO group. At 1 month, the mean difference (MD) was 5.5 points [95% confidence interval (CI): 2.08–8.92, *P* = 0.002], whereas at 3 months, the MD was 6.2 points (95% CI: 2.35–10.05, *P* = 0.002),whereas no significant differences were found at 6- and 12-months postoperatively (*P* > 0.05). The IMN group also achieved significantly higher Lysholm scores at 1 month (MD = 5.7 points, 95% CI: 2.50–8.90, *P* = 0.001) and 3 months (MD = 7.2 points, 95% CI: 3.84–10.56, *P* < 0.001). No statistically significant differences were identified between the groups at 6 and 12 months (*P* > 0.05). For joint ROM, Knee ROM was significantly greater in the IMN group at 1 month (MD = 9.3°, 95% CI: 4.59–14.01, *P* < 0.001) and 3 months (MD = 8.9°, 95% CI: 4.55–13.25, *P* < 0.001), whereas no significant differences were observed at later follow-up points. The ankle ROM showed no significant differences between the two groups at any follow-up time point (*P* > 0.05). Detailed results are presented in Table 3.

**Table 3:** Comparison of functional scores and range-of-motion between the two groups.

Outcome	Time point	IMN group	MIPO group	*t* value	*P* value	Effect size (95% CI)
VAS score	1 month	3.2 ± 0.8	4.1 ± 0.9	4.29	<0.001	MD = −0.9 (−1.31 to −0.49)
	3 months	2.1 ± 0.7	2.8 ± 0.8	3.90	<0.001	MD = −0.7 (−1.06 to −0.34)
	6 months	1.2 ± 0.5	1.4 ± 0.6	1.48	0.143	—
	12 months	0.8 ± 0.4	0.9 ± 0.5	0.96	0.341	—
AOFAS score	1 month	60.3 ± 7.5	54.8 ± 6.9	3.20	0.002	MD = 5.5 (2.08 to 8.92
	3 months	72.5 ± 8.6	66.3 ± 7.9	3.21	0.002	MD = 6.2 (2.35 to 10.05)
	6 months	85.2 ± 6.5	83.1 ± 6.8	1.30	0.197	—
	12 months	92.1 ± 4.3	91.3 ± 4.6	0.78	0.438	—
Lysholm score	1 month	65.4 ± 6.8	59.7 ± 6.2	3.56	0.001	MD = 5.7 (2.50 to 8.90)
	3 months	78.6 ± 7.3	71.4 ± 6.9	4.28	<0.001	MD = 7.2 (3.84 to 10.56)
	6 months	88.9 ± 5.6	86.7 ± 5.9	1.63	0.108	—
	12 months	93.5 ± 4.2	92.6 ± 4.5	0.89	0.377	—
Knee ROM (°)	1 month	105.6 ± 10.2	96.3 ± 9.8	3.94	<0.001	MD = 9.3 (4.59 to 14.01)
	3 months	118.4 ± 9.1	109.5 ± 8.7	4.08	<0.001	MD = 8.9 (4.55 to 13.25)
	6 months	125.7 ± 8.6	122.3 ± 8.9	1.64	0.105	—
	12 months	128.5 ± 8.2	126.7 ± 8.5	0.89	0.376	—
Ankle ROM (°)	1 month	32.5 ± 5.2	31.8 ± 5.6	0.52	0.605	—
	3 months	38.7 ± 4.8	37.9 ± 5.1	0.66	0.510	—
	6 months	42.3 ± 4.2	41.6 ± 4.5	0.67	0.505	—
	12 months	45.1 ± 3.8	44.5 ± 4.0	0.63	0.532	—

Data are presented as mean ± standard deviation. MD, mean difference (IMN group minus MIPO group); CI, confidence interval.

IMN, intermedullary nailing; MIPO, minimally invasive plate osteosynthesis; VAS, Visual Analog Scale; AOFAS, American Orthopedic Foot and Ankle Society; ROM, range-of-motion.

### 3.3 Radiographic evaluation

All patients achieved radiographic bone union. The time to fracture union was significantly shorter in the IMN than in the MIPO group (21.8 ± 2.6 weeks vs. 23.7 ± 2.9 weeks), with a statistically significant difference (t = 2.89, *P* = 0.005). No significant difference was observed between the two groups in terms of postoperative limb length discrepancy (*P* = 0.123). In terms of alignment restoration, postoperative LDTA and ADTA values in both groups were within the normal anatomical range (normal LDTA: approximately 86°–92°; normal ADTA: approximately 78°–82°). Compared with the IMN group, the MIPO group showed values closer to the normal reference range, whereas mild angular deviation was observed in some patients in the IMN group. However, these differences did not reach statistical significance (*P* > 0.05). Detailed results are presented in Table 4.

**Table 4:** Comparison of radiographic outcomes between the two groups.

Group	Time to union (weeks)	LDTA (°)	ADTA (°)	Limb length discrepancy (mm)
IMN group	21.8 ± 2.6	88.1 ± 2.3	79.5 ± 2.7	2.1 ± 0.9
MIPO group	23.7 ± 2.9	89.3 ± 1.8	80.6 ± 2.1	1.8 ± 0.7
*t* value	2.89	1.98	1.92	1.56
*P* value	0.005	0.052	0.058	0.123

Data are given as mean ± standard deviation.

IMN, intermedullary nailing; MIPO, minimally invasive plate osteosynthesis; LDTA, lateral distal tibial angle; ADTA, anterior distal tibial angle.

### 3.4 Complications

During the follow-up period, no cases of deep infection or implant failure were observed in either group. Anterior knee pain occurred in nine patients (25.7%) in the IMN group, which was a significantly greater proportion than that in the MIPO group (2 patients, 5.7%) (*P* = 0.021). Incision-related complications were more common in the MIPO group, occurring in five patients (14.3%; 3 cases of delayed wound healing and 2 cases of superficial infection) compared with only one case (2.9%) in the IMN group. However, this difference did not reach statistical significance (*P* = 0.085). In addition, delayed union was observed in two patients (5.7%) in the IMN group and three patients (8.6%) in the MIPO group, which was not significantly different between the groups (*P* = 0.645). The overall complication rate was 34.3% (12/35) in the IMN group and 28.6% (10/35) in the MIPO group, which was also not statistically significantly different between the groups (*P* = 0.611). Detailed results are presented in Table 5.

**Table 5:** Comparison of postoperative complications between the IMN and MIPO groups.

Complication	IMN group	MIPO group	χ² value	*P* value
Anterior knee pain	9 (25.7%)	2 (5.7%)	5.31	0.021
Delayed union	2 (5.7%)	3 (8.6%)	0.22	0.642
Superficial infection	0 (0.0%)	2 (5.7%)	2.07	0.151
Delayed wound healing	0 (0.0%)	3 (8.6%)	3.11	0.077
Total complications	12 (34.3%)	10 (28.6%)	0.26	0.611

Data are presented as *n* (%). IMN, suprapatellar intramedullary nailing; MIPO, minimally invasive plate osteosynthesis.

### 3.5 Risk factors for postoperative complications

To identify potential risk factors associated with postoperative complications, univariate analysis was performed on the total cohort data, with the occurrence of complications (0 = no, 1 = yes) as the dependent variable. Of the variables analyzed [age, body mass index (BMI), surgical method, fracture classification, operative time, and intraoperative blood loss], surgical method, operative time, fracture classification, and BMI were identified as risk factors for postoperative complications (*P* < 0.05). These variables were subsequently included in a multivariate logistic regression model.

In multivariate analysis, fracture classification, surgical method, operative time, and BMI remained significant independent risk factors for postoperative complications (*P* < 0.05). Specifically, after adjustment for potential confounding factors, MIPO fixation was associated with a higher likelihood of postoperative complications than IMN fixation (OR = 2.87, 95% CI: 1.12–7.36, *P* = 0.028). However, this finding should be interpreted in the context of the nonsignificant difference observed in the crude overall complication rates between the two groups. Additionally, patients with more complex fractures (e.g., AO type 43) had a significantly higher risk of complications than did patients with simpler fractures (OR = 2.64, 95% CI: 1.05–6.63, *P* = 0.039). Furthermore, prolonged operative time and higher BMI were also significantly associated with an increased risk of postoperative complications (*P* < 0.05). Detailed results are presented in Table 6.

**Table 6:** Multivariate logistic regression analysis of risk factors for postoperative complications.

Variable	*β*	SE	Wald	OR	95% CI	*P* value
Surgical method (MIPO)	1.055	0.480	4.82	2.87	1.12–7.36	0.028
Fracture classification (complex)	0.970	0.470	4.25	2.64	1.05–6.63	0.039
Operative time	0.032	0.015	4.51	1.03	1.00–1.06	0.034
BMI	0.118	0.052	5.14	1.13	1.02–1.25	0.023

OR, odds ratio; CI, confidence interval; MIPO, minimally invasive plate osteosynthesis; BMI, body mass index.

## 4 Discussion

Given that distal one-third tibial fractures pose surgical challenges related to anatomy and biomechanics (19–21) and that achieving adequate fracture reduction and stable fixation is difficult, selecting the appropriate strategy for restoring lower limb alignment while preserving the blood supply at the fracture site is critical for optimal management of these injuries (22). In this study, we comprehensively compared the suprapatellar IMN and MIPO fixation strategies in terms of perioperative parameters, functional recovery, radiographic outcomes, and postoperative complications. We demonstrated that IMN involved a shorter operative time, less intraoperative blood loss, and a smaller incision length than did MIPO. The VAS, AOFAS, and Lysholm scores at 1- and 3-months postoperatively, but not from 6-months postoperatively onward, were significantly better with IMN than with MIPO. Compared with the IMN group, the MIPO group demonstrated a tendency toward improved alignment restoration, as reflected by LDTA and ADTA values closer to the normal anatomical range. However, these differences did not reach statistical significance and should therefore be interpreted cautiously. Anterior knee pain was significantly more common with IMN, but incision-related complications were more common with MIPO. We also identified surgical method, operative time, fracture classification, and BMI as independent risk factors for postoperative complications. Although the overall complication rate did not differ significantly between the IMN and MIPO groups, multivariate regression analysis identified an association between MIPO fixation and postoperative complications after adjustment for potential confounding factors. This apparent discrepancy may reflect the influence of fracture complexity, operative time, BMI, and other clinical variables incorporated into the regression model. Therefore, the adjusted association should be interpreted cautiously and should not be considered definitive evidence that MIPO fixation independently increases the overall complication risk.

Our perioperative outcome findings, i.e., that the IMN group had significantly shorter operative time, lower intraoperative blood loss volume, and smaller incision length, suggest that IMN offers advantages in terms of surgical invasiveness and soft tissue preservation. These results were consistent with those of most previous studies, which have reported that IMN, as an intramedullary fixation technique, minimizes the need for extensive soft tissue dissection, thereby reducing intraoperative blood loss and tissue trauma (23). However, some studies have suggested that, with advances in and refinement of MIPO techniques, the differences in surgical invasiveness between the two approaches have gradually diminished (24). Nevertheless, the present study still demonstrated a relative advantage of IMN, indicating that, in real-world clinical practice, certain factors, such as surgeon experience, operative technique, and soft tissue handling, may continue to influence the degree of surgical trauma. Additionally, we found that the number of intraoperative fluoroscopic exposures was lower in the MIPO group. This indicated that IMN in distal tibial fractures relies more heavily on fluoroscopic guidance during guidewire insertion and distal locking, part (25).

Regarding functional recovery, the present study demonstrated that the IMN group achieved significantly better VAS, AOFAS, and Lysholm scores at 1- and 3-months postoperatively, whereas these differences between the groups diminished from 6 months after surgery. This suggests that, although the two techniques yield comparable long-term outcomes, their recovery trajectories differ. A systematic review indicated that previous studies have reported inconsistent findings on this issue (26). Some authors have suggested that MIPO may facilitate better functional recovery due to the absence of direct interference with the knee joint. However, this assumption was not supported by our current results. From a biomechanical perspective, IMN represents a central fixation method that aligns more closely with the physiological load-bearing axis of the lower limb, thereby promoting early weight-bearing and functional recovery (27). In contrast, MIPO, which is an eccentric fixation technique, can provide stable fixation, but its load transmission pathway is less optimal, which may partly explain the relatively slower early recovery. Furthermore, the suprapatellar approach allows the procedure to be performed in a semi-extended knee position, reducing patellar tendon tension and minimizing the need for repeated manipulation. This may decrease intra-articular irritation and could contribute to improved early postoperative knee function, which could partly account for the superior early recovery observed with IMN (28).

In terms of radiographic outcomes, we showed that the time to fracture union was significantly shorter in the IMN than in the MIPO group. While previous studies have reported inconsistent findings on this issue, our results support the notion that IMN may be more favorable for fracture healing, highlighting a clinically meaningful advantage of IMN (29). From a biomechanical perspective, IMN, as a central fixation method, results in more uniform stress distribution and allows controlled micromotion at the fracture site, thereby promoting secondary bone healing. In contrast, although MIPO preserves the periosteal blood supply, its relatively rigid fixation may reduce local mechanical stimulation, potentially affecting callus formation and delaying healing. With respect to alignment restoration, the MIPO group exhibited LDTA and ADTA values closer to the normal anatomical range, which although not statistically significant, suggest a potential advantage of MIPO in maintaining alignment in distal tibial fractures. This trend was consistent with the findings of previous studies that indicated that intramedullary nails may provide less stable distal fixation due to the widened distal medullary canal, which increases the risk of angular deformity, whereas plate fixation allows more precise alignment control (30). Taken together, these findings suggest a trade-off between the biological advantages of IMN in promoting fracture healing and the mechanical advantages of MIPO in achieving optimal alignment.

Regarding complications, we demonstrated that the incidence of anterior knee pain was significantly higher in the IMN group, whereas incision-related complications were more frequent in the MIPO group. These findings were generally consistent with those of previous reports (31). Although the suprapatellar approach has been shown to reduce the incidence of anterior knee pain as compared with the traditional infrapatellar approach, procedure-related discomfort cannot be completely avoided, as the technique involves instrumentation through the knee joint. In the present study, the incidence of anterior knee pain in the IMN group was 25.7%, which was lower than the previously reported rates, suggesting that the suprapatellar approach may offer advantages in minimizing knee joint injury (32). In MIPO, the plate is positioned subcutaneously along the anteromedial aspect of the tibia, where soft tissue coverage is limited, making the region more susceptible to mechanical irritation and increasing the risk of wound-related complications, such as delayed healing and superficial infection.Although the overall complication rate did not differ significantly between the IMN and MIPO groups, multivariate regression analysis suggested an association between MIPO fixation and postoperative complications after adjustment for potential confounders. This apparent discrepancy may be partly explained by differences in fracture complexity, operative time, BMI, and soft tissue conditions, which may have influenced the adjusted model. However, given the relatively small number of complication events, this finding should be interpreted cautiously and should not be regarded as definitive evidence that MIPO was associated with complication risk in the adjusted mode.

Multivariate logistic regression analysis identified the surgical method, operative time, fracture classification, and BMI as independent risk factors for postoperative complications (33). Specifically, the MIPO technique was associated with a higher complication risk, which may be related to local soft tissue conditions and mechanical irritation from subcutaneous plate placement, as indicated above. Prolonged operative time, often reflecting increased surgical complexity, may lead to extended soft tissue exposure and consequently elevate the infection risk. Patients with more complex fractures (e.g., AO type 43) had a significantly higher risk of complications, indicating that fracture complexity is a critical factor in clinical outcomes (21). Furthermore, higher BMI was also associated with an increased risk of complications, potentially due to the compromised local blood supply and increased wound tension, both of which may impair wound healing (34). These findings suggest that surgical decision-making should not rely solely on fracture characteristics, but should also take patient-related factors and perioperative conditions into consideration to optimize individualized treatment strategies. Moreover, IMN in distal tibial fractures still presents challenges in achieving adequate distal fixation stability. Due to the widened distal medullary canal, a mismatch between the nail and cortical bone may occur, leading to reduced distal control and an increased risk of angular deformity. Although recent advances, such as multiplanar distal locking techniques, have partially addressed this issue, careful patient selection remains essential, particularly in cases of comminuted fractures or osteoporosis (30). In contrast, MIPO provides more stable distal support via bridge plating, which contributes to its advantage in maintaining alignment, particularly in distal fractures.

The observed differences among perioperative, functional, radiographic, and complication-related outcomes likely reflect the distinct biomechanical and surgical characteristics of the two fixation techniques. For example, earlier mobilization and less soft tissue disruption associated with IMN may contribute to faster early functional recovery, whereas MIPO may offer improved alignment control through more direct reduction of metaphyseal fragments, albeit potentially at the expense of increased soft tissue irritation. Therefore, interpretation of individual outcomes should consider the interaction between fracture characteristics, surgical strategy, and postoperative rehabilitation.

This study provides a real-world comparative evaluation of suprapatellar IMN and MIPO fixation for distal tibial fractures through a multidimensional assessment of perioperative parameters, functional recovery, radiographic outcomes, and postoperative complications. Nevertheless, several limitations should be acknowledged. First, this was a retrospective single-center study without randomization, and treatment allocation was influenced by fracture characteristics, soft tissue condition, patient-specific factors, and surgeon preference. Therefore, selection bias and confounding by indication cannot be entirely excluded despite comparable baseline characteristics between groups. Although multivariate regression analysis was performed, residual confounding may still exist because propensity score matching or other advanced statistical adjustment methods were not applied. Second, the relatively limited sample size, particularly for complication analysis, may have reduced the statistical robustness of certain findings. In addition, the inclusion of both diaphyseal and metaphyseal distal tibial fractures may have introduced clinical heterogeneity, and subgroup analyses according to fracture classification were not performed because of the limited sample size. Third, repeated-measures analysis was not performed for longitudinal outcomes, and the relatively short follow-up period may have limited assessment of long-term outcomes. Finally, no adjustment for multiple comparisons was performed; therefore, the findings should be interpreted cautiously because of the potential risk of type I error.

In conclusion, both suprapatellar IMN and MIPO fixation are effective treatment options for distal one-third tibial fractures. IMN may offer potential advantages in reducing surgical invasiveness, facilitating early functional recovery, and promoting fracture healing, whereas MIPO may provide better alignment control in selected patients, particularly in distal fractures requiring greater stability, although further prospective studies are warranted. These findings should be interpreted cautiously given the retrospective nature and limited sample size of the present study. These findings indicate that surgical decision-making should be individualized and should take into account fracture characteristics, soft tissue conditions, as well as patient-specific factors.

## Data Availability

The raw data supporting the conclusions of this article will be made available by the authors, without undue reservation.
